# Activity-related dyspnea in older adults participating in the Canadian Longitudinal Study on Aging

**DOI:** 10.1007/s11606-021-07374-4

**Published:** 2022-07-11

**Authors:** Chris P. Verschoor, Sabit Cakmak, Anna O. Lukina, Robert E. Dales

**Affiliations:** 1 Health Sciences North Research Institute, Sudbury, Ontario Canada; 2 Northern Ontario School of Medicine, Sudbury, Ontario Canada; 3 Population Studies Division, Environmental Health Science and Research Bureau, Health Canada, 269 Laurier Ave. West, Ottawa, ON K1A 0K9 Canada; 4 Ottawa Hospital Research Institute, Ottawa Hospital, University of Ottawa, Ottawa, Ontario Canada

**Keywords:** CLSA, Air pollution, Obesity, Depression, Diabetes, Dyspnea

## Abstract

**Background:**

Dyspnea is associated with functional impairment and impaired quality of life. There is limited information on the potential risk factors for dyspnea in an older adult population.

**Objectives:**

Among older adults aged 45 to 85 years of age, what sociodemographic, environmental, and disease related factors are correlated with dyspnea?

**Design:**

We used cross-sectional questionnaire data collected on 28,854 participants of the Canadian Longitudinal Study of Aging (CLSA). Multinomial regression was used to assess the independent effect of individual variables adjusting for the other variables of interest.

**Key Results:**

The adjusted odds ratios for dyspnea “walking on flat surfaces” were highest for obesity (OR, 5.71; 95%CI, 4.71-6.93), lung disease (OR, 3.91; 95%CI, 3.41-4.49), and depression (OR, 3.68; 95%CI, 3.15-4.29), and were greater than 2 for lower income, and heart disease. The effect of diabetes remained significant after adjusting for sociodemographics, heart disease and BMI (OR, 1.61; 95%CI, 1.39-1.86). Those with both respiratory disease and depression had a 12.78-fold (95%CI, 10.09-16.19) increased odds of exertional dyspnea, while the corresponding OR for the combination of heart disease and depression was 18.31 (95%CI, 13.4-25.01).

**Conclusions:**

In a community sample of older adults, many correlates of dyspnea exist which have significant independent and combined effects. These factors should be considered in the clinical context where dyspnea is out of proportion to the degree of heart and lung disease. Whether or not diabetes may possibly be a risk factor for dyspnea merits further investigation.

**Supplementary Information:**

The online version contains supplementary material available at 10.1007/s11606-021-07374-4.

## INTRODUCTION

Dyspnea is an uncomfortable sensation of breathing.^1,2^ Based on the US NHANES 2007-2012 data, 27.7% of those aged 40-79 years old without a diagnosis of chronic obstructive lung disease (COPD) reported having “shortness of breath either when hurrying on the level or walking up a slight hill”.^3^ Dyspnea reduces functional status,^4^ health related quality of living^5^ and is associated with a reduced long-term survival in patients with COPD and heart disease.^6,7^

Understanding the etiology of dyspnea is an important first step towards management. In clinical settings, the diagnostic approach generally focuses on clinical examination and testing to detect cardiac and respiratory diseases.^8^

The Canadian Longitudinal Study on Aging (CLSA) is a population based study which follows the health and lifestyle/activities of older adults.^9^ Using the cross-sectional data from the first evaluation of participants, the present study investigates the role of sociodemographic characteristics, BMI, diabetes, anxiety, and depression and exposure to air pollution as possible risk factors for dyspnea. Information gained by this study should be of value in the management of this common and potentially disabling symptom which requires that we be aware of the many possible risk factors and their relative importance in the population, and to better understand their individual and combined effects.

## METHODS

### Study Population

The CLSA study population is a national stratified random sampling of adults between the ages of 45 and 85 years old at time of recruitment and living in one of the 10 Canadian provinces. Details of the study are described elsewhere.^9^ Beginning in 2011, 51,221 subjects completed an extensive questionnaire-based assessment of their health and social situation The present study focuses on the data from the cross-sectional results of the comprehensive cohort, which is a subgroup of 30,097 who had detailed in-person physical assessments and were asked questions about the presence of dyspnea and comorbidities between May, 2012 and May, 2015.

### Health-related Data

We identified three levels of dyspnea based on questionnaire responses: level 1 (normal), no reports of shortness of breath or shortness of breath only following strenuous activity; level 2, shortness of breath climbing stairs or walking uphill, but no shortness of breath walking on flat surfaces; and level 3, shortness of breath walking on flat surfaces. These questions were similar to those used in the previously validated MRC dyspnea scale.^10^ We excluded 970 participants who answered yes to the question, “Have you had an attack of shortness of breath that came on during the day when you were at rest at any time within the last 12 months?”, as it referred to an episode that could have occurred only once in a year or once in a lifetime, and was therefore, of uncertain clinical significance. Of the remaining, we excluded 273 participants that answered “I don’t know”, had missing data or refused to answer any of the dyspnea questions used in the analysis. Thus, the final sample included in the analysis was 28,854.

Body mass index (BMI) was measured and expressed in kg/m^2^. It was classified as normal (BMI=18.5-24.9), underweight (<18.5), overweight (25 to 29.9) and obese (≥30). Obesity was further categorized as class I (BMI 30-34.9), class II (BMI 35-39.9) and class III (BMI ≥ 40).

Heart disease was defined by a self-report of ever being told by a doctor that the participant has/had any one of heart disease, congestive heart failure, angina, chest pain due to heart disease, a heart attack or myocardial infarction, an unstable heart condition, or required a revascularization procedure. Respiratory disease was defined by a self-report that a doctor had ever told the participant that they had asthma, emphysema, chronic bronchitis, COPD, or “chronic changes in lungs due to smoking”. Anxiety was defined as a physician-diagnosed, “anxiety disorder such as a phobia,

obsessive-compulsive disorder or a panic disorder?” Major depression was defined as a score > 10 on the previously validated 10-item Center for Epidemiologic Studies Depression Scale (CES-D).^11–13^

### Spirometry

TruFlow Easy-On Spirometers were used respecting the ATS/ERS standards for data collection.^14^ Inclusion in the present study required at least three acceptable tests, with ≤ 150ml difference between the 2 best forced vital capacity (FVC) and 2 best forced expiratory volume (FEV_1_) values. The greatest FEV_1_ was used in the present study. Values were expressed as z-scores using the GLI-2012 reference values.^15^ Participants were categorized as above or below the lower limit of normal for FEV_1_ (i.e., z < -1.645). FEV_1_ correlated with the dyspnea questions providing evidence of their validity. Respective z-scores and interquartile range (IQR) for levels 1, 2, and 3 dyspnea were 0.2 (-0.87, 0.43), -0.56 (-1.2, 0.12) and -0.94 (-1.8, -0.18).

### Environmental Factors

#### Ambient air pollution

Particulate matter with a mass median aerodynamic diameter of < 2.5 μm (PM_2.5_), ozone (O_3_) and nitrogen dioxide (NO_2_) were obtained from the Canadian National Air Pollution Surveillance Program (NAPS), which has approximately 300 monitoring stations in about 200 communities across Canada.^16^ For analysis, we used the mean of the 24-hr values for the year prior to the first interview date measured at the NAPS^16^ monitors closest to each participant’s area of residence, identified by a six-digit postal code provided by CLSA and using GIS spatial programming (Arc GIS). Only participants residing within 50 km of a NAPS station were assigned air pollution exposure values. For addresses assigned to more than one monitoring station, the participant’s exposure was estimated by inverse distance weighting of the pollutant data obtained from each station and then averaging the stations values over the period.

#### Statistical Analysis

Variables were expressed as the median and interquartile range (IQR) or count and frequency. Variance across categories was tested by one-way ANOVA (age only), Kruskal-Wallis test, or Chi-square test, where applicable. Associations between each variable and dyspnea were determined by polytomous multinomial logistic regression, with level 1 dyspnea assigned as the base for comparison. We compared level 1 with level 2, and level 1 with level 3. Fully-adjusted multivariable models included age, sex, education, income, smoking, O_3_, SO_2_, NO_2_, BMI, diabetes, lung condition, heart condition, anxiety, and depression. Estimates for age are relative to every 10-year change, and pollution variables are relative to an IQR change. PM_2.5_ was not considered for the multivariable model as it exhibited little variation across the dyspnea categories. To calculate the joint effects of lung or heart disease (i.e., primary factors) with dichotomized secondary factors (i.e., age, BMI, depression, income, sex, or smoking) on the odds of dyspnea, two models were identified, one in which a lung disease by smoking term was included, and another in which heart disease by BMI, heart disease by income and heart disease by smoking interaction terms were included (see supplemental methods for further details). Using these models, adjusted and unadjusted, we calculated the odds ratio (OR) and 95% confidence interval for each joint effect pair from the linear combination of each of the main effects of the primary and secondary factor and the interaction term if it improved the model fit. Analyses were performed using VGAM package in R, version 3.6,^17^ and observations with missing values were excluded from analysis.

## RESULTS

### Descriptive results

**Figure 1.**
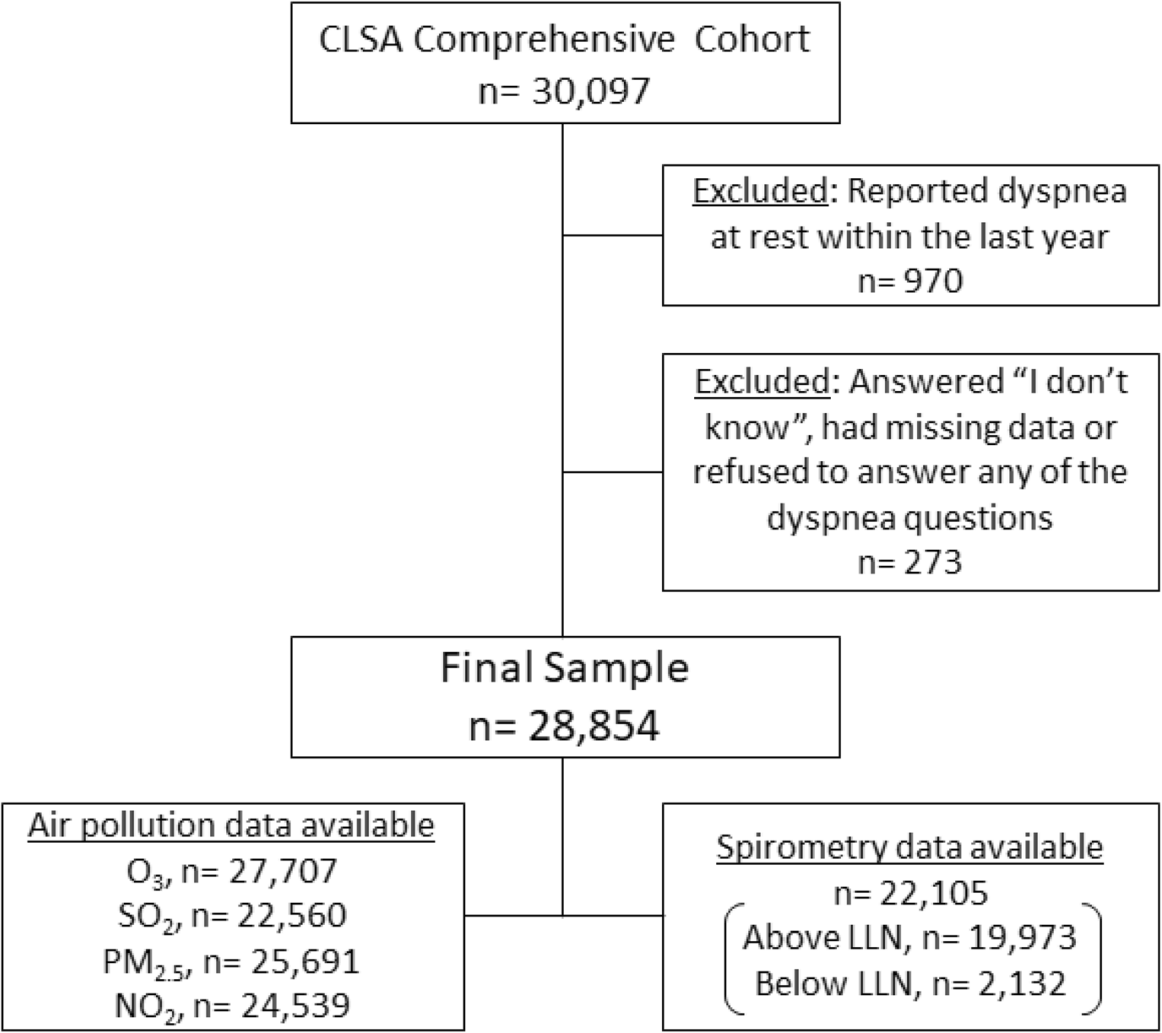
Illustration of how the study sample sizes were derived from the CLSA comprehensive cohort of 30,097.

Data on 28,854 participants were included in the analysis (Figure 1). Twenty six percent of participants reported level 2 or 3 dyspnea. A higher prevalence of dyspnea was associated with older age, female sex, less than post-secondary education, lower income and smoking all p <0.01 (Table 1). Air pollution concentrations were slightly greater in the level 3 group as compared to level 1, with the exception of PM_2.5,_ where the comparison was not significant.

**Table 1.** Percentage or median (interquartile range) of sociodemographic and exposure variables by dyspnea class*

		Dyspnea classification		
Categories	Total	Level 1	Level 2	Level 3
	(N=28,854)	(N=21,280)	(N=5,721)	(N=1,853)
Age (yr.)	62 (54-71)	61 (54-69)	65 (57-74)	67 (60-76)
Sex				
Male	14,260 (49.4%)	11,223 (52.7%)	2,298 (40.2%)	739 (39.9%)
Female	14,594 (50.6%)	10,057 (47.3%)	3,423 (59.8%)	1,114 (60.1%)
Education				
Post-secondary diploma/degree	22,417 (77.7%)	17,120 (80.5%)	4,119 (72.0%)	1,178 (63.6%)
Some post-secondary	2,126 (7.4%)	1,503 (7.1%)	463 (8.1%)	160 (8.6%)
High school diploma	2,719 (9.4%)	1,810 (8.5%)	644 (11.3%)	265 (14.3%)
Less than high school	1,545 (5.4%)	821 (3.9%)	481 (8.4%)	243 (13.1%)
Missing	47 (0.2%)	26 (0.1%)	14 (0.2%)	7 (0.4%)
Income (per $1,000 CAD)				
>100	10,039 (34.8%)	8,365 (39.3%)	1,388 (24.3%)	286 (15.4%)
50 to ⋜ 100	9,539 (33.1%)	7,116 (33.4%)	1,887 (33.0%)	536 (28.9%)
20 to ⋜ 50	6,028 (20.9%)	3,772 (17.7%)	1,632 (28.5%)	624 (33.7%)
< 20	1,405 (4.9%)	730 (3.4%)	427 (7.5%)	248 (13.4%)
Missing	1,843 (6.4%)	1,297 (6.1%)	387 (6.8%)	159 (8.6%)
Smoking status				
Never	13,792 (47.8%)	10,677 (50.2%)	2,444 (42.7%)	671 (36.2%)
Former<10 pyr**	6,729 (23.3%)	5,144 (24.2%)	1,223 (21.4%)	362 (19.5%)
Former10+ pyr	5,883 (20.4%)	3,876 (18.2%)	1,435 (25.1%)	572 (30.9%)
Current<10 pyr	467 (1.6%)	356 (1.7%)	95 (1.7%)	16 (0.9%)
Current10+ pyr	1,911 (6.6%)	1,178 (5.5%)	509 (8.9%)	224 (12.1%)
Missing	72 (0.2%)	49 (0.2%)	15 (0.3%)	8 (0.4%)
Air pollutants				
O_3_ (ppb)	25 (22.6-27)	25 (22.4-26.9)	25 (22.9-27.3)	26 (23-27.4)
Missing	1147 (4.0%)	895 (4.2%)	198 (3.5%)	54 (2.9%)
SO_2_ (ppb)	0.84 (0.31-1.31)	0.83 (0.31-1.27)	0.86 (0.34-1.42)	0.85 (0.307-1.55)
Missing	6,294 (21.8%)	4,349 (20.4%)	1,500 (26.2%)	445 (24.0%)
PM_2.5_ (μg/m^3^)	6.53 (5.81-7.67)	6.53 (5.81-7.65)	6.51 (5.82-7.73)	6.54 (5.79-7.69)
Missing	3,163 (11.0%)	2,393 (11.2%)	547 (9.6%)	223 (12.0%)
NO_2_ (ppb)	8.28 (6.34-10.9)	8.15 (6.33-10.9)	8.54 (6.35-10.9)	8.47 (6.41-10.3)
Missing	4,315 (15.0%)	2,940 (13.8%)	1,049 (18.3%)	326 (17.6%)

*All sociodemographic and exposure variables were associated with variance in dyspnea at p <0.01 except for PM_2.5,_ which was not significant.

**pack-years of cigarette smoking.

To provide evidence of the validity of reported lung disease, we used the sample of 22,105 participants who had information on self-reported lung disease and who had undergone spirometry. The unadjusted FEV_1_ and the FEV_1_ z-scores for those with lung disease were 2.49 L (SD 0.769) and -0.762 (SD 1.12). For those without lung disease, respective values were 2.79 L (SD 0.762), and -0.324 (SD 1.04), with p-values for group differences <0.0001.

All of the comorbidities in Table 2 were associated with dyspnea. The prevalence of level 2 comorbidities was generally intermediate between levels 3 and 1, indicating an ordinal relation. There was an approximate 10-fold difference for the class 3 obesity group, which comprised only 1.4% of the normal group but 13.3% of the level 3 group (Table 2). Depression was over three times more common in the level 3 compared to the level 1 group, 29.9% versus 8.6% (Table 2). Apart from comorbidities, FEV_1_, available for 22,105 participants decreased with increasing dyspnea levels providing evidence of validity of the dyspnea questions. Respective z-scores and IQR for levels 1, 2, and 3 dyspnea were 0.2 (-0.87, 0.43), -0.56 (-1.2, 0.12) and -0.94 (-1.8, -0.18).

**Table 2.** Prevalence percentages of selected comorbidities by dyspnea class *

		Dyspnea classification		
	Total	Level1	Level2	Level3
	(N=28,854)	(N=21,280)	(N=5,721)	(N=1,853)
BMI (kg/m^2^)				
Normal	8,620 (29.9%)	7,251 (34.1%)	1,104 (19.3%)	265 (14.3%)
Underweight	202 (0.7%)	140 (0.7%)	49 (0.9%)	13 (0.7%)
Overweight	11,684 (40.5%)	8,993 (42.3%)	2,131 (37.2%)	560 (30.2%)
Obese-I**	5,525 (19.1%)	3,593 (16.9%)	1,449 (25.3%)	483 (26.1%)
Obese-II	1,839 (6.4%)	964 (4.5%)	607 (10.6%)	268 (14.5%)
Obese-III	905 (3.1%)	298 (1.4%)	361 (6.3%)	246 (13.3%)
Missing	79 (0.3%)	41 (0.2%)	20 (0.3%)	18 (1.0%)
Diabetes				
No	23,804 (82.5%)	18,267 (85.8%)	4,315 (75.4%)	1,222 (65.9%)
Yes	5,003 (17.3%)	2,979 (14.0%)	1,399 (24.5%)	625 (33.7%)
Missing	47 (0.2%)	34 (0.2%)	7 (0.1%)	6 (0.3%)
Lung disease				
No	24,190 (83.8%)	18,589 (87.4%)	44,70 (78.1%)	1,131 (61.0%)
Yes	4,517 (15.7%)	2,612 (12.3%)	1,209 (21.1%)	696 (37.6%)
Missing	147 (0.5%)	79 (0.4%)	42 (0.7%)	26 (1.4%)
Heart disease				
No	24,660 (85.5%)	18,832 (88.5%)	4,577 (80.0%)	1,251 (67.5%)
Yes	3,911 (13.6%)	2,283 (10.7%)	1,072 (18.7%)	556 (30.0%)
Missing	283 (1.0%)	165 (0.8%)	72 (1.3%)	46 (2.5%)
Anxiety				
No	26,416 (91.6%)	19,757 (92.8%)	5,084 (88.9%)	1,575 (85.0%)
Yes	2,385 (8.3%)	1,488 (7.0%)	625 (10.9%)	272 (14.7%)
Missing	53 (0.2%)	35 (0.2%)	12 (0.2%)	6 (0.3%)
Depression				
No	25,355 (87.9%)	19,402 (91.2%)	4,667 (81.6%)	1,286 (69.4%)
Yes	3,427 (11.9%)	1,834 (8.6%)	1,039 (18.2%)	554 (29.9%)
Missing	72 (0.2%)	44 (0.2%)	15 (0.3%)	13 (0.7%)

*All comorbidities were associated with variance in dyspnea at p <0.01.

**Class I (BMI 30-34.9), Class II (BMI 35-39.9) and Class III (BMI ≥ 40).

### Adjusted results

**Figure 2.**
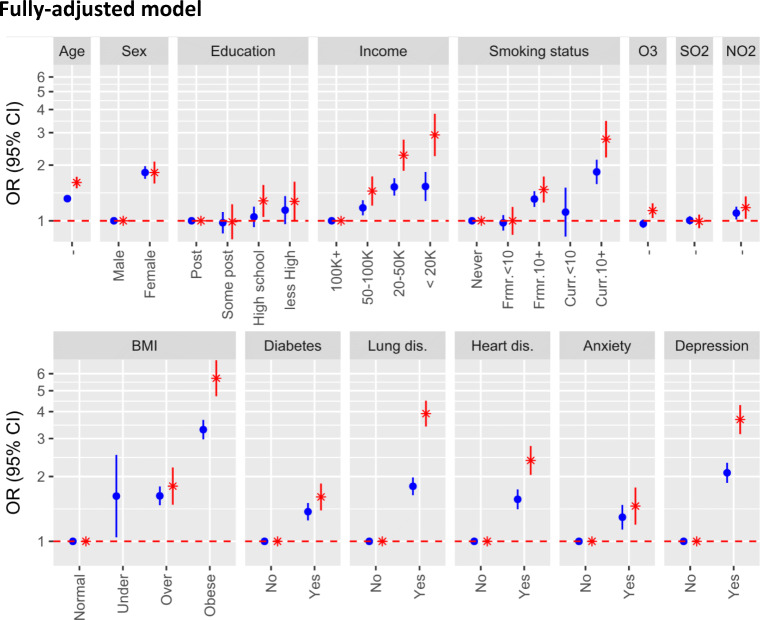
Adjusted odds ratios from a multinomial regression model comparing dyspnea levels 2 and 3 to level 1. The model was adjusted for all sociodemographic factors, exposures, and comorbidities considered (excluding PM_2.5_), and the odds ratio (OR) and 95% confidence interval (CI) for the level 2 versus level 1 (blue circles) and level 3 versus level 1 (red asterisks) comparisons are presented. Note, estimates for age are relative to every decade change, and O_3_, SO_2_ and NO_2_ are relative to an IQR increase. For BMI, level 3 versus 1 is not plotted for underweight people due to low numbers.

The adjusted OR of being at level 3 dyspnea was highest for obesity, 5.71 (95%CI, 4.71-6.93), lung disease, 3.91 (95%CI, 3.41-4.49), and depression, 3.68 (95%CI, 3.15-4.29) and were greater than 2 for lower income, current smoking status and heart disease (Figure 1). The small effects of O_3_ and NO_2_ remained (OR, 1.14; 95%CI, 1.04-1.24 and OR, 1.18; 95%CI, 1.03-1.36, respectively). Notably, the association of diabetes remained significant after adjusting for all covariates, including sociodemographic, heart disease and BMI (OR, 1.61; 95%CI, 1.39-1.86) (Figure 2).

Odds ratios for level 2 dyspnea were generally less than for level 3 dyspnea and excluded one for all variables except education, SO_2_ and O_3_.

### Selected joint effects in those with reported heart or lung disease

For lung disease, the largest joint effects were with BMI and depression with unadjusted OR for level 3 dyspnea of 16.44 (95%CI, 14.21-19.01) and 18.29 (95%CI, 15.66-21.36), respectively (Table 3). The corresponding unadjusted OR for level 2 dyspnea were much smaller at 4.61 (95%CI, 4.17-5.08) and 4.39 (95%CI, 3.93-4.91) (Table 3). Adjusted OR for these comparisons were smaller but the confidence intervals overlapped with the unadjusted values, indicating that they were not statistically significantly different.

**Table 3.** Odds ratios (95%CI) for the joint effects of reported heart or lung disease plus the presence of a secondary risk indicator for dyspnea in unadjusted and adjusted models. * The comparator is the absence of both the primary factor and the higher risk category of the secondary factor

		Level 2 vs Level 1 dyspnea		Level 3 vs Level 1 dyspnea	
Primary factor	Secondary factor	Unadjusted	Adjusted	Unadjusted	Adjusted
Lung disease	Age ≥63 yr.	3.43 (3.11, 3.78)	2.61 (2.26, 3.01)	11.26 (9.69, 13.08)	7.28 (5.79, 9.16)
	BMI >30 kg/m^2^**	4.61 (4.17, 5.08)	3.98 (3.46, 4.58)	16.44 (14.21, 19.01)	12.44 (9.99, 15.49)
	Depression	4.39 (3.93, 4.91)	3.52 (3.01, 4.13)	18.29 (15.66, 21.36)	12.78 (10.09, 16.19)
	Income <$50,000**	1.91 (1.76, 2.06)	1.69 (1.5, 1.9)	4.36 (3.9, 4.86)	3.41 (2.86, 4.08)
	Female sex	3.07 (2.79, 3.38)	2.98 (2.59, 3.42)	6.81 (5.92, 7.84)	6.15 (4.95, 7.65)
	Smoking ≥10 pack-years**	2.68 (2.35, 3.06)	2.85 (2.45, 3.32)	7.45 (6.01, 9.24)	7.56 (6.21, 9.21)
Heart disease	Age ≥63 yr.	2.68 (2.44, 2.93)	3.52 (2.96, 4.18)	5.95 (5.20, 6.80)	4.9 (3.74, 6.42)
	BMI >30 kg/m^2^**	5.3 (4.63, 6.07)	4.21 (3.46, 5.12)	19.84 (15.78, 24.93)	10.26 (7.69, 13.67)
	Depression	4.67 (4.16, 5.25)	4.78 (3.93, 5.83)	17.3 (14.69, 20.39)	18.31 (13.4, 25.01)
	Income <$50,000**	2.01 (1.8, 2.23)	2.3 (1.95, 2.71)	4.1 (3.49, 4.82)	4.9 (3.74, 6.42)
	Female sex	3.92 (3.52, 4.38)	4.07 (3.36, 4.92)	8.59 (7.28, 10.13)	8.89 (6.49, 12.19)
	Smoking ≥10 pack-years**	3.43 (3, 3.93)	2.56 (2.09, 3.12)	8.59 (7.28, 10.13)	7.13 (5.3, 9.58)

*unadjusted multinomial regression models included only the primary and secondary factors and an interaction term if applicable. Adjusted models includes those factors, along with all other factors in the main effects model and interaction terms.

**an interaction term with the primary factor was included when modelling.

For heart disease, the largest joint effects were also with BMI and depression with unadjusted OR for level 3 dyspnea of 19.84 (95%CI, 15.78-24.93) and 17.3 (95%CI, 14.69-20.39), respectively (Table 3). Corresponding unadjusted OR for level 2 dyspnea were over 2/3 smaller at 5.3 (95%CI, 4.63-6.07) and 4.67 (95%CI, 4.16-5.25) (Table 3). Adjusted OR for these comparisons were smaller, but the confidence intervals overlapped with the unadjusted values except for the OR of the joint effect of obesity and heart disease, 10.49 (95%CI, 7.84-14.03).

To validate the joint effects found with reported lung disease, the analysis was repeated substituting reported lung disease by FEV1 z-scores dichotomized as less than the lower limits of normal (LLN) or not (Table 4). Compared to reported lung disease, the point estimates for odds ratios were greater for the joint effects for level 3 dyspnea using FEV_1_ but the 95%CI overlapped with reported lung disease. Further details about joint effects can be found in the appendix (Supplementary Data Table 1).

**Table 4.** Adjusted odds ratios (95%CI) derived from multinomial regression models for the joint effects of reduced FEV_1_, expressed as z-scores less than the lower limit of normal (LLN) plus the presence of a secondary risk indicator on dyspnea in adjusted models. The comparator is the absence of both the primary and the higher risk category of the secondary factor

Primary factor	Secondary factor	Level 2 vs Level 1	Level 3 vs Level 1
FEV1 < LLN	Age ≥63 yr.	3.26 (2.68, 3.97)	9.34 (6.76, 12.93)
	BMI >30 kg/m^2^	5.11 (4.22, 6.19)	17.35 (12.7, 23.69)
	Depression	4.75 (3.84, 5.88)	19.17 (13.76, 26.71)
	Income <$50,000	2.08 (1.75, 2.47)	4.37 (3.36, 5.7)
	Female Sex	3.88 (3.19, 4.72)	9.9 (7.17, 13.67)
	Smoking ≥10 pack-years)*	2.73 (2.25, 3.31)	7.93 (6.14, 10.24)

Adjusted models included primary and secondary factors and an interaction term if applicable plus all other factors in the main effects model and interaction terms.

*A smoking * FEV_1_ interaction was included as a covariate for all other interaction models because it improved the model fit.

## DISCUSSION

Dyspnea is common among older adult Canadians with approximately one in five participants in our study reporting shortness of breath climbing stairs or walking uphill, and one in fifteen experiencing shortness of breath walking on flat surfaces. In addition to the commonly recognized causes of dyspnea, heart and lung disease, we found several less commonly reported correlates of dyspnea, and our study provides novel information about the strong joint observed effects between selected factors. There is relatively little general population-based information on the sociodemographic correlates of dyspnea. We found that age and total annual household income were strongly related to dyspnea. Similarly, data from 5,473 participants in the 2006/2007 South Australian Health Omnibus population study revealed greater levels of dyspnea in older age groups, females, and those with lower education and income.^18^ It has been theorized that the effect of lower social status on health may be explained by undesirable health related behaviours, access to health care, and comorbidities.^19^ However, our study adjusted for smoking and comorbidities, and income-related differential access to medical care should be minimized by Canada’s national public health care system.

Ozone and NO_2_ were significantly associated with dyspnea but the effects were relatively small. Ambient air pollution has been associated with increased morbidity and mortality from respiratory and cardiac diseases, ^20,21^ but there are few population-based studies addressing air pollution and dyspnea in older adults.^22–24^ The Swiss population based SAPALDIA cross-sectional study comprised of 9,651 adults between 18 and 60 years old found that a 10 μg/m^3^ increase in NO_2_, PM_10_ and O_3_ increased the observed risk of being “troubled by shortness of breath when hurrying on level ground or walking up a slight hill” by 8.5% (95%CI, 3.2, 14.1), 31.6% (95%CI, 18.2, 46.4) and -3.7% (95%CI, -11.3, -4.6), respectively.^22^

Our finding of an increased prevalence of dyspnea in females was similar to what was observed in a large survey representative of adults at least 40 years in major metropolitan regions of Latin America that investigated the prevalence of COPD.^6,23^ Cory et al. (2015) provided evidence supporting qualitative sex differences in the expression of dyspnea.^25^ Our study demonstrated that the sex-related difference in depression was unlikely to be due to differences in underlying social status, heart or lung disease, or depression, which are more common in women than men.^26^

Major depression as defined by the CES-D had a stronger observed effect size on dyspnea than did reported heart disease and was similar in magnitude to lung disease. A European longitudinal study of 542 subjects recruited between 20 and 45 years old also reported that the incidence of dyspnea was related to depression, OR 12.2 (95%CI, 3.97-37.5).^27^ Dyspnea appears to be one of the many somatic complaints associated with depression,^28^ but the mechanisms explaining somatic symptoms are not well understood.^29^ It is also possible that lung disease could precipitate both dyspnea and depression.^30^ Our study contributes to the literature by suggesting that dyspnea on exertion could be a primary symptom of depression and anxiety given that the association persisted after accounting for reported heart and lung disease. We also report the novel finding of a large observed joint effect for the combination of depression with reported heart or lung disease in a population-based study.

In the BOLD study the fully adjusted OR between obesity and dyspnea was 1.92 (95%CI, 1.71-2.15), ^24^ and in the PLATINO study the OR per 1 kg/m^2^ increase in BMI was 1.06 (95%CI, 1.04-1.07) unadjusted for heart disease.^23^ Obesity is associated with a reduced functional residual capacity which would tend to narrow the airways and increase airflow resistance. Increased weight around the chest reduces total thoracic compliance and increases the work of breathing. These factors increase the oxygen cost of breathing and predispose to dyspnea on exertion ^32^.

In addition to the direct physiologic effects of obesity,^31^ dyspnea may be related to comorbidities such as cardiac disease and mood disorders, which are more prevalent in individuals who have obesity, 31,^32^ but are often not considered in studies of obesity and breathlessness. Our findings are novel in that they took into account these many plausible confounding variables. Our study is also unique in demonstrating the large observed effect of an increased BMI in those with reported heart or lung disease.

Associations have been reported between diabetes and diagnosed lung diseases, ^33^ but our findings are novel in that we found an association between dyspnea and diabetes adjusting for many variables including BMI, age, sex, income, education, depression, and reported physician diagnosed heart and lung disease in a population-based sample. Hypothetical reasons for this association include endothelial and myocardial dysfunction ^34^ and a diabetic pro-inflammatory state involving the lungs.^33^ Diabetes associated peripheral and autonomic neuropathy, and peripheral vascular disease could also limit exercise tolerance.^35,36^ A unique finding was the very large odds of level 3 dyspnea in those with depression combined with either reported heart or lung disease. Our data suggests that when clinically assessing possible causes of dyspnea, joint effects of risk factors should be considered. Patients with a greater severity of dyspnea than would be expected for a given severity of lung or heart disease may be harbouring other risk factors.

### Strengths and Limitations

Reporting bias is a concern with questionnaire-ascertained data but should have been minimized by the questions for diabetes, and heart disease prefaced by, “Has a doctor ever said you had….” Body mass index was objectively measured and depression was defined by a validated questionnaire. Our dyspnea scale was not previously standardized, but contained questions similar to those found in in the validated and widely used MRC dyspnea scale,^37^ and dyspnea was associated with level of measured lung function in our study population.^10^ Evidence exists to support the validity of self-reported heart disease. Machon et al. (2012) reported a sensitivity of 97.7% and a positive predictive value of 60.7% for self-reported acute myocardial infarction validated by hospital medical records.^38^ Bergmann et al. (1998) found that for subjects with > 12^th^ grade education, the true positive rate was 91% for a self-reported hospitalization for ischemic heart disease confirmed by medical records.^39^ For self-reported diabetes, concordance with a government health database in Ontario Canada was good (kappa 0.8).^40^ There may be other unmeasured and possibly confounding risk factors for dyspnea that we could not examine such as the frequency and intensity of aerobic physical exercise. Malnutrition among those with COPD could also exacerbate dyspnea but we would not expect this situation to be frequent in a population-based study. ^41^

## CONCLUSION

Although cardiac and respiratory diseases are commonly recognized correlates of dyspnea in older adults, consideration should be given to the many individual and combined roles of sociodemographic factors and comorbidities such as depression, obesity and diabetes, which when combined may possibly increase the probability of experiencing dyspnea.

## Supplementary information

ESM 1 (DOCX 20 kb)

## Data Availability

Data are available from the Canadian Longitudinal Study on Aging (www.clsa-elcv.ca) for researchers who meet the criteria for access to de-identified CLSA data.

## **Abbreviations**

AIC: Akaike information criterion
BMI: body mass index
CI: confidence intervals
CLSA: The Canadian Longitudinal Study on Aging
COPD: chronic obstructive pulmonary disease
FEV: forced expiratory volume
FVC: forced vital capacity
IQR: interquartile range
LLN: lower limits of normal
NAPS: Canadian National Air Pollution Surveillance Program
NO_2_: nitrogen dioxide
O_3_: ozone
OR: odds ratio
PM_2.5_: particulate matter with a mass median aerodynamic diameter of < 2.5 micrometers
ppb: parts per billion
SO_2_: sulfur dioxide
μg/m^3^: micrograms per cubic meter of air.
