# Supplementary material for: Activity-related dyspnea in older adults participating in the Canadian Longitudinal Study on Aging
Source: J Gen Intern Med. 2022 Jul 11;37(13):3302–9. doi: 10.1007/s11606-021-07374-4 (PMC9550921; doi:10.1007/s11606-021-07374-4)
Supplement: Supplementary file 1 — (DOCX 20 kb) [file 11606_2021_7374_MOESM1_ESM.docx]

**APPENDIX**

**Supplemental Methods:**

**Study population.** Briefly, participants were recruited using several sampling frames: Statistics Canada’s Canadian Community Health Survey on Healthy Aging, provincial health registries, and random digit dialing telephone sampling. Exclusion factors were significant cognitive impairment, being in the Canadian Armed Forces, living in a long-term care residence, one of the three Canadian territories or on a First Nations reserve. Beginning in 2011, and every three years thereafter, 51,221 subjects completed extensive questionnaire-based assessment of their health and social situation. Participants in the comprehensive cohort, which this study is based, resided within 25 to 50 km of one of 11 physical data collection sites in 7 of the 10 provinces. The interviewer-administered questionnaires and the details of all physical assessments can be found on the CLSA website (<https://www.clsa-elcv.ca/researchers/data-support-documentation>).

**Multinomial regression.** For building the multivariable model, each of the covariates were first entered into separate models including age and sex and coefficients were estimated. Each covariate was then entered into a single model with age and sex in a forward step-wise fashion, and coefficient estimates were compared to that of the initial model. Collinearity was monitored by the variance inflation factor, and the final model including all covariates was observed to exhibit the lowest Akaike information criterion (AIC).

**Interaction analysis.** To calculate the joint effects of lung or heart disease (i.e., primary factors) with age (<63 or ≥63 years old), BMI (obese or not), depression as defined by the Center for Epidemiologic Studies Depression Scale score (depressed or not), income (<50K or ≥50K), sex, or smoking (<10 or ≥10 pack-years) (i.e., secondary factors) on the odds of dyspnea, a step-wise approach was used to identify which interaction terms between heart or lung disease and the secondary factors improved the AIC and residual deviance of a multinomial regression model that included all of the aforementioned factors in addition to the factors included in the final main effects model.

**Supplementary Table S1.** Multinomial regression model used to calculate combined effects for lung disease with selected risk factors.

| **Contrast** | **Predictors** | **Odds Ratios** | **CI** | **p** |
| --- | --- | --- | --- | --- |
| L2 v L1 | (Intercept) | 0.06 | 0.04 – 0.09 | **<0.001** |
|  | Age [63+] | 1.54 | 1.43 – 1.67 | **<0.001** |
|  | Sex [Female] | 1.76 | 1.63 – 1.91 | **<0.001** |
|  | Education [some Post] | 1.02 | 0.89 – 1.17 | 0.75 |
|  | Education [High] | 1.12 | 0.99 – 1.28 | 0.074 |
|  | Education [less High] | 1.22 | 1.03 – 1.45 | **0.024** |
|  | Income [<50K] | 1.46 | 1.34 – 1.59 | **<0.001** |
|  | Smoking [10+] | 1.4 | 1.28 – 1.54 | **<0.001** |
|  | O_3_ | 0.97 | 0.92 – 1.02 | 0.254 |
|  | SO_2_ | 1.01 | 0.96 – 1.06 | 0.773 |
|  | NO_2_ | 1.09 | 1.00 – 1.18 | **0.04** |
|  | BMI [Obese] | 2.36 | 2.18 – 2.55 | **<0.001** |
|  | Diabetes [Yes] | 1.44 | 1.31 – 1.58 | **<0.001** |
|  | Lung.dis [Yes] | 1.69 | 1.50 – 1.90 | **<0.001** |
|  | Heart.dis [Yes] | 1.67 | 1.50 – 1.85 | **<0.001** |
|  | Anxiety [Yes] | 1.28 | 1.13 – 1.46 | **<0.001** |
|  | Depression [Yes] | 2.09 | 1.87 – 2.32 | **<0.001** |
|  | Smoking [10+] * Lung.dis [Yes] | 1.21 | 0.99 – 1.47 | 0.066 |
| L3 v L1 | (Intercept) | 0 | 0.00 – 0.00 | **<0.001** |
|  | Age [63+] | 2.13 | 1.86 – 2.45 | **<0.001** |
|  | Sex [Female] | 1.8 | 1.57 – 2.07 | **<0.001** |
|  | Education [some Post] | 1.06 | 0.86 – 1.32 | 0.579 |
|  | Education [High] | 1.42 | 1.17 – 1.73 | **<0.001** |
|  | Education [less High] | 1.45 | 1.14 – 1.85 | **0.003** |
|  | Income [<50K] | 2.02 | 1.76 – 2.32 | **<0.001** |
|  | Smoking [10+] | 1.57 | 1.32 – 1.85 | **<0.001** |
|  | O_3_ | 1.13 | 1.03 – 1.24 | **0.007** |
|  | SO_2_ | 0.99 | 0.91 – 1.08 | 0.818 |
|  | NO_2_ | 1.16 | 1.01 – 1.34 | **0.033** |
|  | BMI [Obese] | 3.64 | 3.20 – 4.16 | **<0.001** |
|  | Diabetes [Yes] | 1.7 | 1.47 – 1.96 | **<0.001** |
|  | Lung.dis [Yes] | 3.41 | 2.86 – 4.08 | **<0.001** |
|  | Heart.dis [Yes] | 2.61 | 2.24 – 3.04 | **<0.001** |
|  | Anxiety [Yes] | 1.46 | 1.20 – 1.78 | **<0.001** |
|  | Depression [Yes] | 3.75 | 3.21 – 4.36 | **<0.001** |
|  | Smoking [10+] * Lung.dis [Yes] | 1.42 | 1.07 – 1.87 | **0.015** |
|  | Observations | 20695 | | |
|  | Deviance | 24963.43 | | |
|  | AIC | 25035.43 | | |

Estimates are shown for Level 2 dyspnea (L2) relative to Level 1 (L1) and Level 3 (L3) relative to L1. For each categorical factor, the category being compared to the reference is shown in square brackets. Interactions terms are presented as two factors separated by an asterisk.

**Supplementary Table S1 (cont.).** Multinomial regression model used to calculate combined effects for heart disease with selected risk factors.

| **Contrast** | **Predictors** | **Odds Ratios** | **CI** | **p** |
| --- | --- | --- | --- | --- |
| L2 v L1 | (Intercept) | 0.06 | 0.04 – 0.08 | **<0.001** |
|  | Age [63+] | 1.53 | 1.41 – 1.66 | **<0.001** |
|  | Sex [Female] | 1.77 | 1.64 – 1.91 | **<0.001** |
|  | Education [some Post] | 1.02 | 0.90 – 1.17 | 0.723 |
|  | Education [High] | 1.12 | 0.99 – 1.28 | 0.076 |
|  | Education [less High] | 1.23 | 1.04 – 1.47 | **0.018** |
|  | Income [<50K] | 1.54 | 1.40 – 1.69 | **<0.001** |
|  | Smoking [10+] | 1.54 | 1.41 – 1.69 | **<0.001** |
|  | O_3_ | 0.97 | 0.92 – 1.02 | 0.249 |
|  | SO_2_ | 1.01 | 0.96 – 1.06 | 0.759 |
|  | NO_2_ | 1.08 | 1.00 – 1.17 | **0.049** |
|  | BMI [Obese] | 2.46 | 2.26 – 2.68 | **<0.001** |
|  | Diabetes [Yes] | 1.44 | 1.32 – 1.59 | **<0.001** |
|  | Lung.dis [Yes] | 1.8 | 1.64 – 1.98 | **<0.001** |
|  | Heart.dis [Yes] | 2.3 | 1.95 – 2.71 | **<0.001** |
|  | Anxiety [Yes] | 1.28 | 1.12 – 1.45 | **<0.001** |
|  | Depression [Yes] | 2.08 | 1.87 – 2.32 | **<0.001** |
|  | Income [<50K] * Heart.dis [Yes] | 0.75 | 0.61 – 0.93 | **0.007** |
|  | BMI [Obese] * Heart.dis [Yes] | 0.74 | 0.60 – 0.92 | **0.005** |
|  | Smoking [10+] * Heart.dis [Yes] | 0.72 | 0.58 – 0.89 | **0.002** |
| L3 v L1 | (Intercept) | 0 | 0.00 – 0.00 | **<0.001** |
|  | Age [63+] | 2.09 | 1.82 – 2.41 | **<0.001** |
|  | Sex [Female] | 1.81 | 1.58 – 2.08 | **<0.001** |
|  | Education [some Post] | 1.06 | 0.86 – 1.32 | 0.573 |
|  | Education [High] | 1.41 | 1.16 – 1.72 | **0.001** |
|  | Education [less High] | 1.46 | 1.15 – 1.87 | **0.002** |
|  | Income [<50K] | 2.29 | 1.96 – 2.68 | **<0.001** |
|  | Smoking [10+] | 1.86 | 1.59 – 2.17 | **<0.001** |
|  | O_3_ | 1.13 | 1.03 – 1.24 | **0.008** |
|  | SO_2_ | 0.99 | 0.91 – 1.08 | 0.802 |
|  | NO_2_ | 1.16 | 1.01 – 1.33 | **0.035** |
|  | BMI [Obese] | 4.28 | 3.67 – 4.98 | **<0.001** |
|  | Diabetes [Yes] | 1.7 | 1.47 – 1.96 | **<0.001** |
|  | Lung.dis [Yes] | 3.91 | 3.41 – 4.49 | **<0.001** |
|  | Heart.dis [Yes] | 4.9 | 3.74 – 6.42 | **<0.001** |
|  | Anxiety [Yes] | 1.45 | 1.19 – 1.77 | **<0.001** |
|  | Depression [Yes] | 3.74 | 3.21 – 4.35 | **<0.001** |
|  | Income [<50K] * Heart.dis [Yes] | 0.6 | 0.45 – 0.81 | **0.001** |
|  | BMI [Obese] * Heart.dis [Yes] | 0.5 | 0.37 – 0.67 | **<0.001** |
|  | Smoking [10+] * Heart.dis [Yes] | 0.78 | 0.58 – 1.05 | 0.105 |
|  | Observations | 20695 | | |
|  | Deviance | 24922.008 | | |
|  | AIC | 25002.008 | | |

Estimates are shown for Level 2 dyspnea (L2) relative to Level 1 (L1) and Level 3 (L3) relative to L1. For each categorical factor, the category being compared to the reference is shown in square brackets. Interactions terms are presented as two factors separated by an asterisk.
